# Ultra-Small Iron Nanoparticles Target Mitochondria Inducing Autophagy, Acting on Mitochondrial DNA and Reducing Respiration

**DOI:** 10.3390/pharmaceutics13010090

**Published:** 2021-01-12

**Authors:** Lorenzo Rivas-García, José Luis Quiles, Alfonso Varela-López, Francesca Giampieri, Maurizio Battino, Jörg Bettmer, María Montes-Bayón, Juan Llopis, Cristina Sánchez-González

**Affiliations:** 1 Biomedical Research Centre, Department of Physiology, Institute of Nutrition and Food Technology, Faculty of Pharmacy, University of Granada, Avda. del Conocimiento s/n, 18071 Armilla, Spain; lorenrivas@ugr.es (L.R.-G.); jlquiles@ugr.es (J.L.Q.); alvarela@ugr.es (A.V.-L.); jllopis@ugr.es (J.L.); 2 Sport and Health Research Centre, University of Granada, C/. Menéndez Pelayo 32, 18016 Armilla, Spain; 3 Centre for Nutrition and Health, Universidad Europea del Atlántico (UEA), 39011 Santander, Spain; 4 Department of Clinical Specialist and Odontostomatological Sciences (DISCO)-Sez, Biochemistry, Polytechnic University of Marche, 60131 Ancona, Italy; f.giampieri@staff.univpm.it (F.G.); m.a.battino@staff.univpm.it (M.B.); 5 Department of Biochemistry, Faculty of Sciences, King Abdulaziz University, Jeddah 21589, Saudi Arabia; 6 International Research Center for Food Nutrition and Safety, Jiangsu University, Zhenjiang 212013, China; 7 Department of Analytical Chemistry, Faculty of Chemistry and Instituto de Investigación Sanitaria del Principado de Asturias (ISPA), University of Oviedo, 33007 Oviedo, Spain; bettmerjorg@uniovi.es (J.B.); montesmaria@uniovi.es (M.M.-B.)

**Keywords:** nanotechnology, mitochondria, respiration, mtDNA deletions, copy number, metals

## Abstract

The application of metallic nanoparticles (materials with size at least in one dimension ranging from 1 to 100 nm) as a new therapeutic tool will improve the diagnosis and treatment of diseases. The mitochondria could be a therapeutic target to treat pathologies whose origin lies in mitochondrial dysfunctions or whose progression is dependent on mitochondrial function. We aimed to study the subcellular distribution of 2–4 nm iron nanoparticles and its effect on mitochondrial DNA (mtDNA), mitochondrial function, and autophagy in colorectal cell lines (HT-29). Results showed that when cells were exposed to ultra-small iron nanoparticles, their subcellular fate was mainly mitochondria, affecting its respiratory and glycolytic parameters, inducing the migration of the cellular state towards quiescence, and promoting and triggering the autophagic process. These effects support the potential use of nanoparticles as therapeutic agents using mitochondria as a target for cancer and other treatments for mitochondria-dependent pathologies.

## 1. Introduction

Nanotechnology is referred to as the area of science focused on the study of the synthesis, characterization, and application of materials and functional systems of particles with sizes ranging between 1 and 100 nm. Nowadays, the interest in these materials is not only due to their small size, but also at these dimensions the material properties change in comparison to the same material at the macroscopic scale and make possible highly specific cellular and molecular interactions. The application of nanotechnology to biomedical research (nanobiotechnology) will improve, in the next few years, the development of new and more effective tools for the detection and treatment of diseases like diabetes, Parkinson’s disease, or cancer [1,2]. This science will progress the monitorization, reparation, and control of biological functions by using systems on a nanoscale. Despite the development of nanoparticles for different biomedical applications, the exposition to certain types of particles could promote cell damage. Nowadays, the accepted paradigms of the toxicity of nanomaterials are oxidative stress and inflammation. In fact, our research group described the damage produced by Au (gold) nanoparticles in DNA structure, ROS production, and lipid in vitro and in vivo conditions and its relation with the nanoparticle size [3], but other mechanisms are still not well-defined.

Iron oxide nanoparticles are widely used in biomedical research, especially in cancer research, due to their properties of magnetism, biocompatibility and due to their drug delivery and multi-imaging functions [4]. Even the treatment of anemia using iron nanoparticles have been developed in the last years to reduce the side effects of traditional treatments due to the reduced size of nanoparticles that could increase its absorption [5]. Moreover, iron nanoparticles, especially the iron oxide, have been developed as an alternative in gene therapy and as a drug delivery system [6]. Recent studies have been conducted to evaluate some more specific and relevant aspects related to toxicity mechanisms of iron oxide nanoparticles like mitochondrial damage, ROS production or autophagy [7]. Khan, M.I. and co-workers [8] showed that certain iron oxide nanoparticles promoted cell death in lung cancer cells through ROS production and autophagy, but these nanoparticles did not promote cell death in normal lung cells. On the other hand, other authors have proposed the role of iron nanoparticles in autophagosome induction and oxidative damage in mammary gland cells [9]. Nonetheless, more studies are necessary on the possible interaction between iron nanoparticles and mitochondria. Autophagy is an intracellular degradation process that delivers cytoplasmic constituents to lysosomes for degradation in response to a high variety of stimuli [10]. This process is related to create new cellular structures [11], so it represents a catabolic process of cytosolic renovation, but it is also able to induce autophagy-dependent cell death [12]. There are defined three types of autophagy depending on how the autophagic substrate move to the lysosome: macro-autophagy, micro-autophagy, and chaperone-mediated autophagy. One of the effects of iron nanoparticles could be associated with the induction of autophagy due to most endocytic routes of nanomaterial cell uptake by phagocytic and non-phagocytic mechanisms converge upon the lysosome.

In the present study, the effect of ultra-small iron nanoparticles on autophagy induction and mitochondrial function are evaluated using an in vitro model. Two types of ultra-small iron nanoparticles have been tested, a commercial preparation used in clinical practice (Venofer^®^) and one synthesized in the laboratory (FeNPs). This work addresses, for the first time, a comprehensive study about the traffic of iron and other elements in the subcellular fractions, the integrity of the mtDNA, the mitochondrial functionality and the induction of the autophagy process that will increase the knowledge about the metabolism of nanoparticles and its possible application in therapy focused on mitochondria. To perform the study HT-29 cell line was chosen based on previous studies in which the implications of nanomaterials in therapy have been evaluated [13,14,15].

## 2. Materials and Methods

### 2.1. Synthesis and Characterization of Nanoparticles: Synthesis of Ultra-Small Nanoparticles (FeNPs)

Iron nanoparticles (4 nm core Fe_2_O_3_ coated with tartaric/adipic acid) were synthesized following a slightly modified protocol from Pereira et al. [16]. This method is based on the precipitation of Fe^3+^ in the presence of highly basic medium (5 mol L^−1^ NaOH solution) with the addition of tartrate and adipic acid solution for the iron core coating as described somewhere else [15]. The molar ratio tartaric: adipic: Fe used corresponds to 1:1:2, which has given best performance in previous experiments. The three components are mixed and constantly stirred in a buffer media (ammonium acetate 50 mmol L^−1^ at pH 4). The initial pH of the mixture is increased stepwise until reaching pH 8. When mixture turns dark brown/blackish, centrifugation and ultrafiltration (30,000 Da; 3000 Da Ultra-15 MWCO centrifugal filter units, Millipore, Darmstadt, Germany) steps are needed to separate the microparticulate and nanoparticulate iron fractions from the supernatant and remove excess of soluble ligands and the rest of reagents. Centrifuge Biofuge Stratos Heraeus (Thermo Scientific^TM^, Waltham, MA, USA) was used for these purposes. Size and shape characterization of the particles has been conducted by TEM, DLS, and UV-VIS. The characterization of these nanoparticles was published in previous articles of our research group [15].

### 2.2. Cell Culture Conditions

HT-29 cell lines were obtained from the Cell Culture Resource Centre at the University of Granada, Spain. The cells were precultured in 25 cm^2^ culture flasks in RPMI-1640 medium supplemented with 10% (*v*/*v*) FBS and 2 mmol L^−1^ L-glutamine. The culture flasks were maintained in a cell incubator at 37 °C in a humidified atmosphere of 5% CO_2_ and 95% air. Medium was replaced every 2–3 days after rinsing with PBS. Upon reaching confluence, cells were treated with trypsin-EDTA solution (Sigma, St Louis, MO, USA) and split 1/10 to allow for continuous growth. Cells were exposed to 0.5 mmol L^−1^ Fe NPs concentration over 48 h. Cells cultured were treated with 0.5 mmol L^−1^ FeNPs or Venofer^®^ for 48 h. Every week, the absence of mycoplasma was evaluated employing a commercial kit (Sigma, St Louis, MO, USA).

### 2.3. TEM Analysis

Once the exposure time had finished, cells were prepared to be visualized by TEM. Cells samples were fixed with fresh primary fixative (1.5% glutaraldehyde, 1.0% formaldehyde in 0.05 mol L^−1^ sodium cacodylate buffer, pH 7.4) and post-fixed with secondary fixative (1% osmium tetroxide, 1% potassium ferrocyanide in Milli Q water) followed by dehydration with ascending series of alcohol before embedding samples in epoxy resin. Ultra-thin sections were cut and doubly stained with uranyl acetate and lead citrate. A transmission electron microscope LIBRA 120 PLUS microscope at 120 kV (Carl Zeiss SMT, Oberkochen, Germany) was used to determine the distribution of ultra-small iron nanoparticles.

### 2.4. Subcellular Fractionation

HT-29 cells were previously washed with physiologic saline solution. Mitochondria isolation was carried out employing a commercial kit according to the manufacturer’s instruction (Abcam, Cambridge, UK). Briefly, cells were trypsinized, collected by centrifugation and frozen. Then, cells were homogenized and centrifuged at 1000× *g*, 10 min, 4 °C, two times. Both supernatants were collected and the pellet with the nucleus portion was preserved. Afterward, the supernatants were centrifuged at 12,000× *g* during 10 min, 4 °C, obtaining the pellet with the isolated of mitochondria. Supernatant was also preserved in which ERP, Golgi, and cytosol was included.

### 2.5. Determination of Total Metal Concentrations

Determination of sodium (Na), magnesium (Mg), phosphorus (P), potassium (K), calcium (Ca), iron (Fe), copper (Cu), manganese (Mn), zinc (Zn) and selenium (Se) in subcellular fractions, diluted with a basic solution containing ammonium hydroxide, butanol, EDTA, and triton X-100, was performed by means an ICP-MS instrument (Agilent 7500, Agilent Technologies, Tokyo, Japan) fitted with a Meinhard type nebulizer (Glass Expansion, Romainmotier, Switzerland) and equipped with a He collision cell. A Milli-Q system (Millipore, Bedford, MA, USA) was used to obtain deionized water (18 MΩ cm). All reagents (ammonium hydroxide solution, butanol, EDTA, Triton X-100) used were of the highest available purity. A standard solution of 100 µg L^−1^ of Li, Mg, Sc, Co, Y, In, Ce, Ba, Pb, Bi, and U in 1% (*v*/*v*) HNO_3_ was prepared from a 1.000 mg L^−1^ multi-element stock standard solution (Merck, Darmstadt, Germany) and used for daily optimizing of the ICP parameters. Single-element standard solutions for ICP-MS containing 1.000 µg mL^−1^ of each analyte were also purchased from Merck. Calibration curves were prepared using Ga as an internal standard and by the dilution of stock solutions of 1.000 mg L^−1^ in 1% HNO_3_. The accuracy of this method was evaluated by comparison with a certified reference material Seronorm^TM^ Trace Elements Serum (Billingstad, Norway) and by recovery studies of spiked samples with multi-element standards. The calculated recoveries for each element were between 95% and 105% in all cases. For each element, we used the mean of five separate determinations.

### 2.6. Proportion of Deleted mtDNA and mtDNA Copy Number Assessment

The proportion of deleted mtDNA was determined using a quantitative reverse-transcription polymerase chain reaction (QRT-PCR) to amplify two mitochondrial genes, MT-ND4 and MT-ND1. Relative levels of mtDNA copy number were also determined by QRT-PCR by amplifying the nuclear single-copy nuclear gene acidic ribosomal phosphoprotein PO (36B4) and using the amplification data of mitochondrial MT-ND1 gene previously obtained. DNA amplification was performed in a MicroAmp Optical 384-well Reaction Plate (Applied Biosystems, Foster City, CA, USA) using the Applied Biosystem’s 7900HT Fast Real-Time PCR system. Primers for the assays were as follows: MT-ND1 forward primer 5′-CCCTAAAACCCGCCACATCT′-3, MT-ND1 reverse primer GAGCGATGGTGAGAGCTAAGGT-3′, MT-ND4 forward primer 5′-CCATTCTCCTCCTATCCCTCAAC-3′, MT-ND4 reverse primer 5′-CACAATCTGATGTTTTGGTTAAACTATATTT-3′, 36B4 forward primer 5′-CTGCAGATTGGCTACCCGAC-3′ and 36B4 reverse primer 5′-CACAGACAAAGCCAGGACCC-3′. Final primer concentrations were 200 nmol L^−1^ except for the last two that were 300 and 500 nmol L^−1^, respectively. Reactions were performed in 10 µL volumes. Reaction mixture in each well included genomic DNA (5 ng), 2× QuantiNova SYBR Green PCR Master Mix (Applied Biosystems, Foster City, CA, USA) (5 μL), ROX dye (Applied Biosystems, Foster City, CA, USA) (1 μL), Mili-Q sterilized H_2_O and the proper primer oligonucleotides. Reaction conditions were set at 95 °C for 2 min followed by 40 cycles of data collection consisting of a denaturation step at 95 °C for 8 s and an annealing/extension at the 58 °C step for 15 s. An additional phase to obtain a melting curve was performed at the end of each reaction to verify specific amplification. Standard curves were included for analysis of data. Mean quantification cycle (Cq) for each sample were calculated using Sequence Detector Systems version 2.4 software. The proportion of deleted mtDNA was calculated for each well dividing MT-ND4 by MT-ND1 amplification data taking into account reaction efficiency values. The resulting values were divided by the average value obtained from control samples (untreated cells) analysis. This allowed to obtain a relative MT-ND4:MT-ND1 ratio. In turn, the MT-ND1:36B4 ratio was calculated and multiplied by two to obtain mtDNA copy number for each sample. DNA used in the aforementioned assays was isolated using a NucleoSpin Tissue kit (Macherey-Nagel GmbH & Co. KG, Düren, Germany) according to manufacturers.

### 2.7. Determination of Energetic Metabolism

#### 2.7.1. Mitochondrial Respiration

The oxygen consumption rate (OCR) in HT-29 cells was measured in real-time using a XF-24 Extracellular Flux Analyzer (Seahorse Bioscience, Billerica, MA, USA) as previously reported [17]. For this, 3 × 10^4^ cells were seeded for 16 h in the XF-24 plate and treated for 48 h on 0.5 mmol L^−1^ of FeNPs or VENOFER^®^. The medium was replaced with 450 μL/well of XF-24 running media (Seahorse Bioscience, Billerica, MA, USA), supplemented with 25 mmol L^−1^ glucose, 2 mmol L^−1^ glutamine, 1 mmol L^−1^ sodium pyruvate, without serum and pre-incubated at 37 °C for 20 min in the XF Prep Station incubator (Seahorse Bioscience, Billerica, MA, USA) in the absence of CO_2_. The plate was then transferred to the XF-24 Extracellular Flux Analyzer, and after an OCR baseline measurement, four sequential injections of compounds that affect bioenergetics were performed, as follows: 55 μL of oligomycin (1 μg mL^−1^), 61 μL of 2,4-Dinitrophenol (2,4 DNP) (1 mmol L^−1^), and 68 μL of antimycin A/rotenone (10 μmol L^−1^/1 μmol L^−1^) at injection in port C. Each treatment was carried out in three replicates and the final results were expressed as pmol of O_2_ consumed per 105 cells per minute (pmol O_2_/105 cells/min).

#### 2.7.2. Glycolysis

To evaluate glycolysis, extracellular acidification rate (ECAR) was measured in real-time using a XF-24 Extracellular Flux Analyzer (Seahorse Bioscience, Billerica, MA, USA) as previously reported [17]. ECAR was measured after addition of 55 μL of rotenone (1 μmol L^−1^), 61 μL of glucose (30 mmol L^−1^) and 68 μL 2-Deoxy-d-glucose (2-DG) (100 mmol L^−1^). Prior to measure ECAR, HT-29 cells were treated as in previous assay per triplicate and average ECAR value, expressed as milli-pH per minute (mpH/min) per 3.0 × 10^4^ cells, was calculated for each treatment.

### 2.8. Autophagy

#### 2.8.1. Autophagy Induction

The CYTO-ID ENZKIT175 Autophagy Detection Kit 2.0 (Enzo Life Sciences, Lausen, Switzerland) was used for the detection of autophagy in HT-29 cells. Briefly, HT-29 cells were seeded at a density of 0.75 × 10^5^ cells/mL and left to adhere for 24 h. Then, cells were treated with 0.5 mM FeNPs for 48 h, untreated (control group) or rapamycin 0.5 µmol L^−1^ (autophagy inductor) for 12 h. Then, the supernatant was removed and the cells were washed twice with 200 µL of PBS supplemented with 5% (*v*/*v*) FBS and stained with an autophagy staining solution containing CYTO-ID^®^ Green Detection Reagent and Hoechst 33342 Nuclear Stain following the protocol provided by the manufacturer. Fluorescence was determined using a microplate reader (Biotek, VT, USA), excitation 480 nm-emission 530 nm and also a confocal fluorescence microscope. For confocal fluorescence microscopy, staining solution was removed after 30 min of incubation at 37 °C in a humidified incubator, and cells were washed with 200 µL of PBS supplemented with 5% (*v*/*v*) FBS three times. Then, cells were fixed with PFA 4% (*v*/*v*), at room temperature in the absence of light for 20 min, and washed with 200 µL of PBS supplemented with 5% (*v*/*v*) FBS three times. Then microscope slide preparation with glycerol and cells were visualized and photographed in a confocal microscopy (Nikon, Tokyo, Japan) with a coupled camera (ref) Nis-Elements microscope imaging software (version 4.13).

#### 2.8.2. Autophagic Flux

The levels of p62 were assessed in cells by the p62 Elisa kit (Enzo Life Sciences, Lausen, Switzerland) according to the manufacturer’s protocol. Briefly, after 48 h exposure to iron nanoparticles and rapamycin 0.5 µmol L^−1^ (as positive control), cells were collected and protein were extracted and seeded on a pre-coated plate with p62 antibody. After incubating samples in presence of a second anti-p62 antibody (rabbit polyclonal), the amount of p62 was revealed by adding a secondary donkey anti-rabbit IgG antibody conjugated to horseradish peroxidase and a mix composed by TMB and hydrogen peroxide. The plate was read in a microplate reader (Biotek, Winooski, VT, USA) and the amount of p62 normalized for the total protein amount.

### 2.9. Apoptosis/Necrosis

To quantify if nanoparticles induced apoptosis or necrosis in HT-29 cells, a double staining with annexin V and propidium iodide was performed. Cells were seeded in 35 mm culture dishes (VWR, Radnor, PA, USA) at 2 × 10^5^ cells/mL and incubated in the conditions described in Section 2.2 Culture medium was removed after 24 h and replaced with 1 mL of fresh medium containing either the IC50 of extracts or 0.4 µM of doxorubicin (positive control). Cells were incubated for 48 h, collected by trypsinization and centrifuged at 1000× *g* for 5 min at room temperature (RT). The obtained pellet was rinsed twice with 1 mL of cold PBS 1× intercalated with 5 min of centrifugations at 1000× *g*. Following centrifugations, 100 μL of annexin binding buffer 1×, 5 μL of annexin V-FITC and 2 μL of PI (Annexin V-FITC Apoptosis Detection Kit; Invitrogen, Waltham, MA, USA) was added to all samples and incubated for 15 min at RT in the absence of light. Afterwards, to these cellular suspensions were added 400 μL of annexin binding buffer 1× and 500 μL of cold PBS 1×. The analysis and quantification of apoptotic events and necrosis were performed by flow cytometry on Attune Acoustic Focusing Flow Cytometer (Life Technologies, Carlsbad, CA, USA) using an Attune Cytometric software (Life Technologies), with the acquisition of at least 10,000 events per sample. Data presented as the mean of two independent experiments.

### 2.10. Statistical Analysis

Descriptive statistical parameters (means and standard deviations of 8 samples for each experiment) were obtained for each of the variables studied. Statistical comparisons among the groups were performed by a comparison of means between independent variables. All analyses were performed using SPSS 20.0 (SPSS, Chicago, IL, USA). Differences were considered statistically significant at a probability level <5%.

## 3. Results

### 3.1. TEM Images

**Figure 1:**
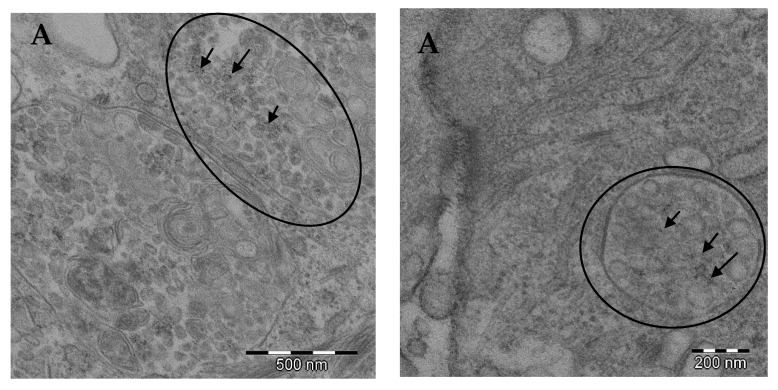

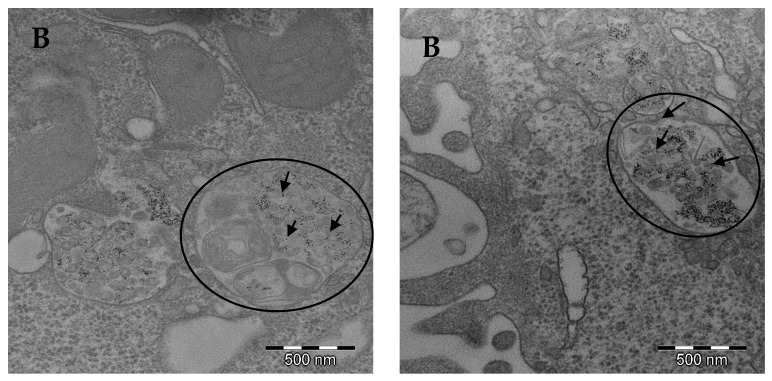
TEM images of cultured cells HT-29 exposed to (**A**) 0.5 mmol L^−1^ 4 nm iron nanoparticles (FeNPs) and (**B**) 0.5 mmol L^−1^ Venofer^®^. Ellipses highlight the presence of mitophagy processes. Arrows highlight the presence of nanoparticles.

Nanoparticles administered to the cells were observed by transmission electron microscopy (TEM). As shown in Figure 1, the presence of autolysosomes was found after 48 h of exposure. This finding is compatible with the mitophagy process. No differences for the presence of nanoparticles nor for the formation of autophagosomes were found between the synthesized iron nanoparticles and the commercial form (Venofer^®^, Figure 1) administered.

### 3.2. Total Metal Distribution

Table 1 shows the results of total metal concentration. The results showed that at basal conditions, the highest levels of iron were found in the mitochondria fraction. When cells were exposed to ultra-small FeNPs, the highest increase in the iron levels was also found for the fraction corresponding to mitochondria. The methodology employed did not discern if mitochondria are free of lysosomes, thus some lysosomal metals could be included in this fraction.

On the other hand, in relation to the rest of the elements studied, at basal conditions, the Mg levels found were significantly lower in the fraction corresponding to the nucleus, the highest levels of Ca and Se were registered in the fraction corresponding to the nucleus followed by the fraction corresponding to the mitochondria. Na and K levels were significantly higher in cytosol than in other subcellular fractions. The levels of Cu in the fraction corresponding to the nucleus were significantly higher than those found in the cytosol.

After exposing the cells to ultra-small FeNPs, there was an increase in all the other elements studied in the fraction corresponding to the mitochondria, except for Cu. In the nucleus, an increase in Cu levels was registered as a consequence of exposure to nanoparticles.

### 3.3. Proportion of Deleted mtDNA and mtDNA Copy Number

**Figure 2:**
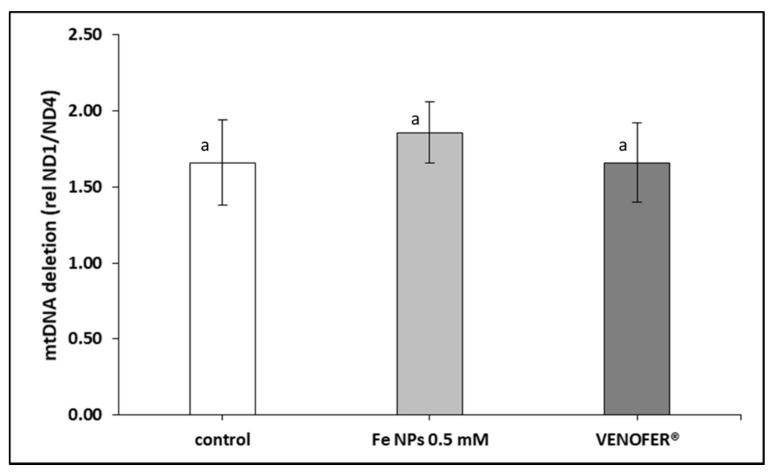
ND1/ND4 mtDNA deletion. Results expressed as mean ± SEM. (*p* < 0.05). (a) vs. control (*p* < 0.05).

**Figure 3:**
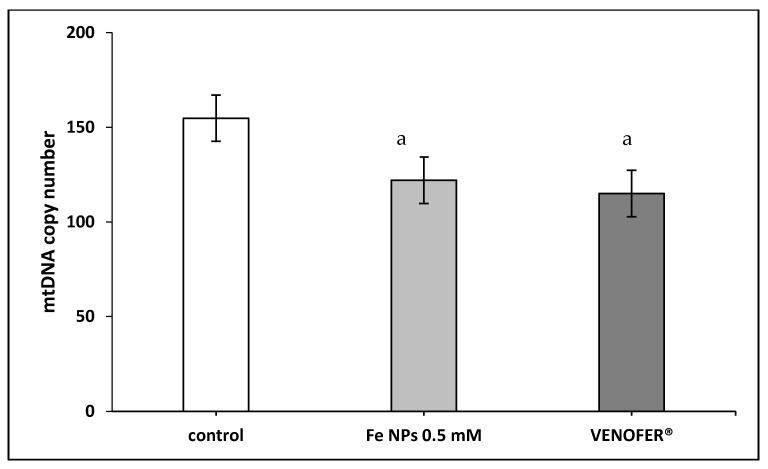
ND1/ND4 mtDNA copy number. Results expressed as mean ± SEM. (a) vs. control (*p* < 0.05).

Figure 2 show the proportion of mtDNA deleted in HT-29 cells. No significant differences were found in this parameter between groups. However, when colorectal cells were treated with both types of iron nanoparticles, mtDNA copy number was lower (Figure 3).

### 3.4. Determination of Energetic Metabolism

**Figure 4:**
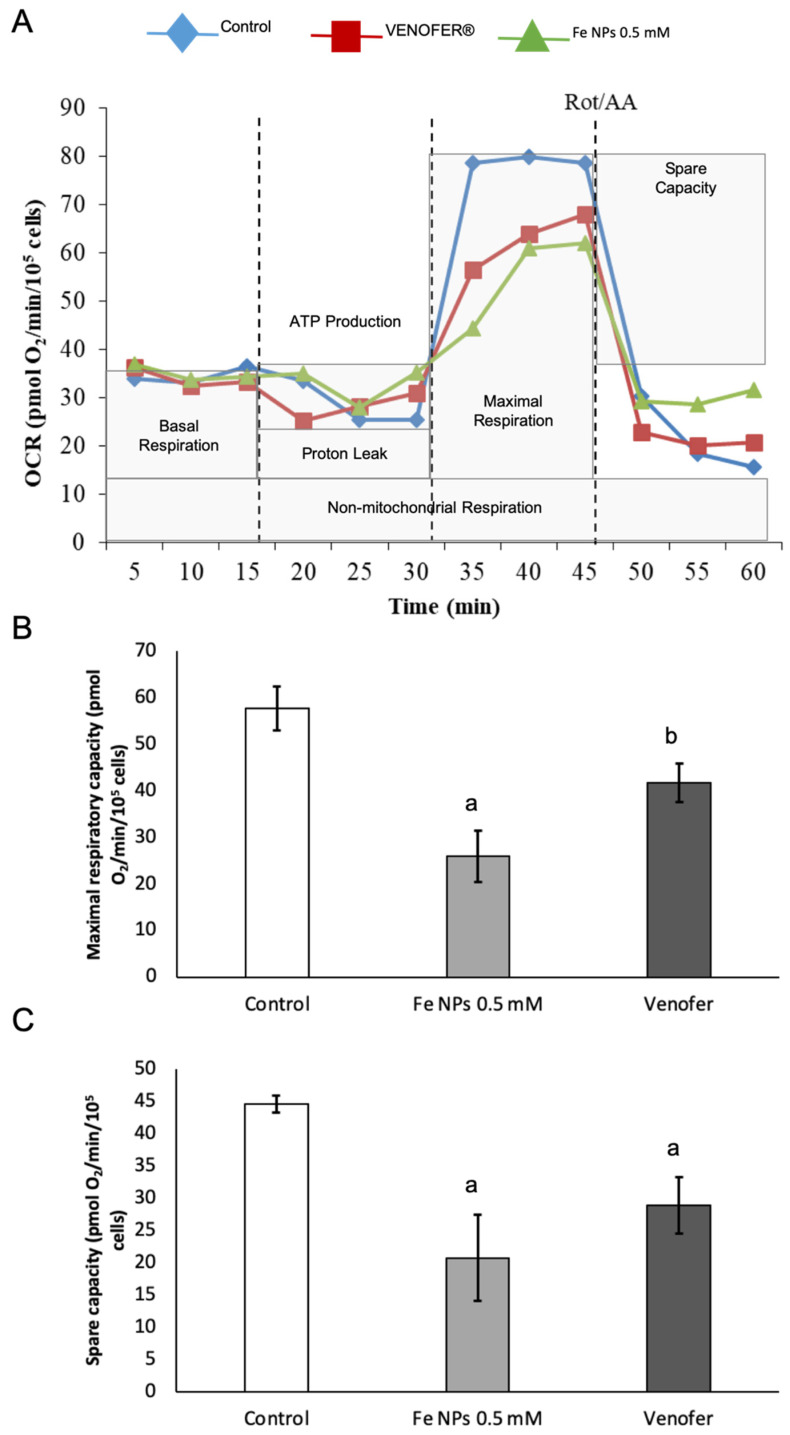
Modulation of mitochondrial respiration by iron nanoparticles in HT-29 cells. Cells were treated with DMEM (ctrl), iron nanoparticles Fe NPs or Venofer^®^ for 24 h. OCR was determined by using the Seahorse XF-24 Extracellular Flux Analyzer after the sequential injections of oligomycin (1 μg mL^−1^), 2,4-DNP (1 mmol L^−1^), and rotenone/antimycin (1 μmol L^−1^/10 μmol L^−1^). (**A**) shows OCR. (**B**) shows maximal respiratory capacity. (**C**) shows the spare capacity. Data are indicated as the mean ± SEM (*n* = 3). (a) vs. control; (b) vs. Fe NPs 0.5 mM (*p* < 0.05).

The effects of nanoparticles on mitochondrial functionality were assessed firstly by analyzing OCR by using Agilent Seahorse XF24 Analyzer (Figure 4). Measured parameters were basal respiration (which shows energetic demand of the cell under baseline conditions), ATP production (that shows ATP produced by the mitochondria that contribute to meeting the energetic needs of the cell), proton leak (that can be a sign of mitochondrial damage or can be used as a mechanism to regulate the mitochondrial ATP production), maximal respiration (that shows the maximum rate of respiration that the cell can achieve) and spare capacity (that represents the cell’s ability to respond to demand, and that can be an indicator of cell fitness flexibility). Differences were found for maximal respiration and spare capacity, with the highest value found for control group.

**Figure 5:**
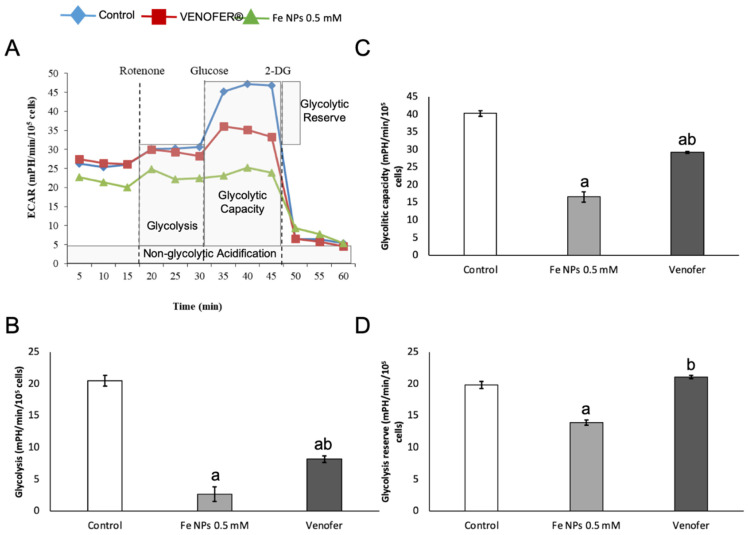
Effects on Mitochondrial glycolysis by iron nanoparticles in HT-29 cells. Cells were treated with DMEM (ctrl), iron nanoparticles Fe NPs or Venofer^®^ for 24 h. ECAR was determined by using the Seahorse XF-24 Extracellular Flux Analyzer after the injections of rotenone (1 μmol L^−1^), glucose (30 mmol L^−1^) and 2-DG (100 mmol L^−1^). Glycolytic capacity was calculated from the XF glycolysis stress test profile. (**A**) shows ECAR. (**B**) shows glycolysis. (**C**) shows the glycolitic capacity. (**D**) shows the glycolysis reserve. Data are indicated as the mean ± SEM (*n* = 3). (a) vs. control; (b) vs. Fe NPs 0.5 mmol L^−1^ (*p* < 0.05).

**Figure 6:**
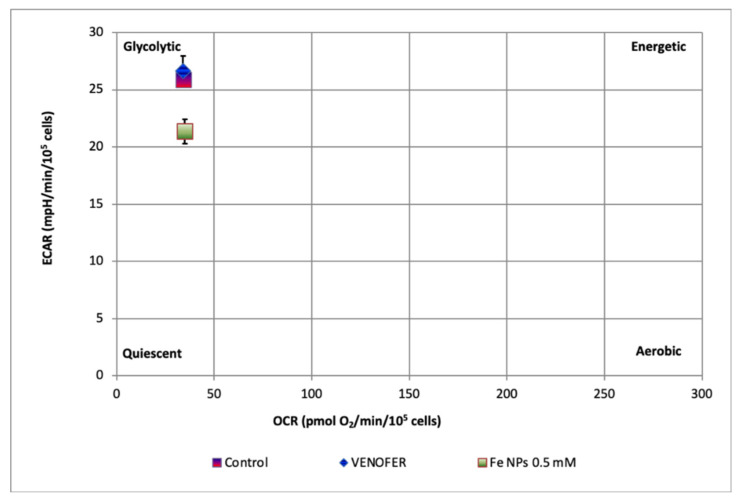
ECAR:OCR ratio plot showing the metabolic phenotype of HT-29 cells treated or not with iron nanoparticles FeNPs or Venofer^®^. Data represent the mean ± SEM (*n* = 3).

Concerning glycolysis, three parameters were measured (Figure 5), namely glycolysis (presented as the ECAR reached by a given cell after the addition of saturating amounts of glucose), glycolytic capacity (that represents the cell’s use of glycolysis to its maximum capacity) and glycolytic reserve (that indicates the capability of a cell to respond to an energetic demand as well as how close the glycolytic function is to the cell’s theoretical maximum). For the three parameters, the iron nanoparticles led to lower values than control and Venofer^®^ groups. Treatment of cells with Venofer^®^ led to lower values than control group only for glycolysis and glycolytic capacity. Metabolic phenotype was calculated as the ECAR:OCR ratio (Figure 6). The most relevant observation was that Fe NPs slightly moved cells from a net glycolytic phenotype to a more quiescent state.

### 3.5. Autophagy

**Figure 7:**
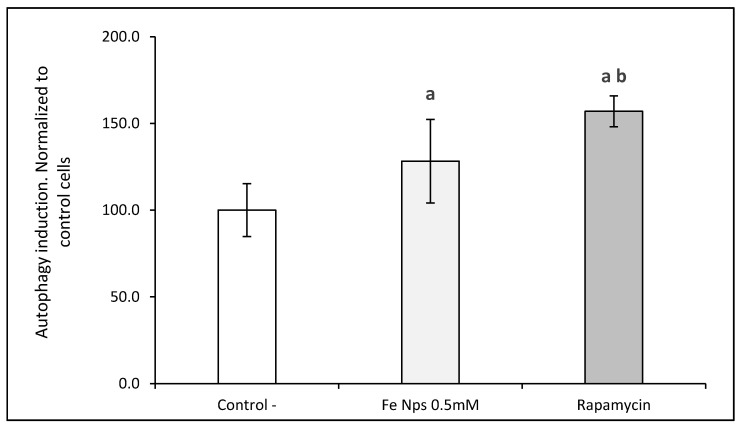
Autophagy induction evaluated by fluorescence using a microplate reader in HT-29 normalized to control cells (untreated). 5 mmol L^−1^, rapamycin 0.5 µmol L^−1^ and control cells (untreated). Results expressed as mean ± SEM. (a) vs. control; (b) vs. Fe NPs 0.5 mmol L^−1^ (*p* < 0.05).

**Figure 8:**
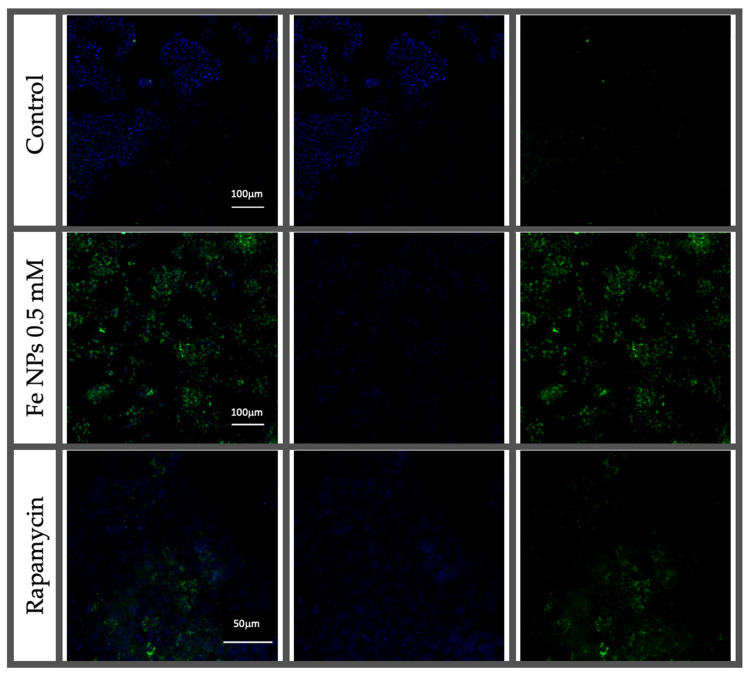
Confocal microscopy images of HT-29 cells exposed to Fe NPs 0.5 mmol L^−1^, rapamycin 0.5 µmol L^−1^ and control cells (untreated).

The assessment of autophagy in HT-29 cells was analyzed to verify whether iron nanoparticles induced this process. For this purpose, fluorescence signal was performed using two dyes: Hoechst 33342 dye, which is a cell permeable nucleic acid dye and Cyto-ID^®^ Green dye, a 488 nm excitable green fluorescent reagent, which becomes brightly fluorescent in vesicles produced during autophagy (Enzo Life Sciences, Lausen, Switzerland) and normalized with respect to the signal produces by non-treated cells. Treatment of HT-29 cells with FeNPs led to higher content of autophagic vacuoles with respect to those found in control group, but lower than those found in cells treated with the inductor of autophagy (Figure 7). Moreover, in confocal microscopy study, cells treated with FeNPs displayed increased fluorescence in comparison to unexposed cells indicating higher content of autophagic vacuoles. These results confirmed that the exposure of cells to FeNPs induced autophagy process in HT-29 colorectal cells (Figure 8).

**Figure 9:**
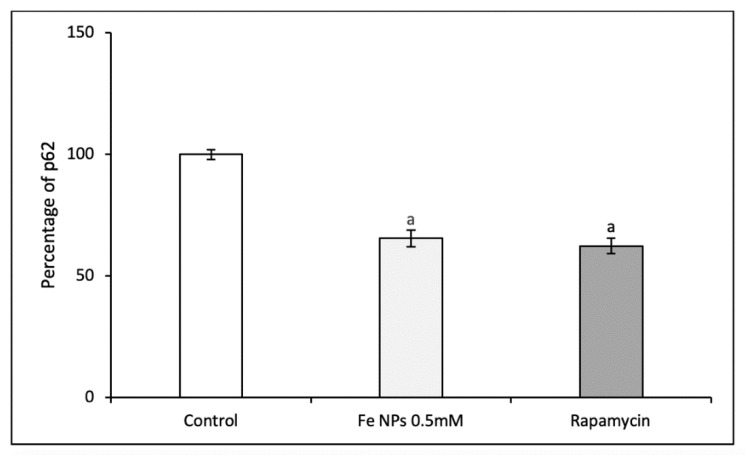
Percentage of p62 (respect control cells) in HT-29 cells exposed to Fe NPs 0.5 mmol L^−1^, rapamycin 0.5 µmol L^−1^. Results expressed as mean ± SEM. (a) vs. control (*p* < 0.05).

On the other hand, Figure 9 shows that the incubation with FeNPs or rapamycin reduced significantly the quantity of p62 protein with respect to control cells, providing information about that the autophagic flux was initiated by the FeNPs exposition.

### 3.6. Apoptosis/Necrosis

**Figure 10:**
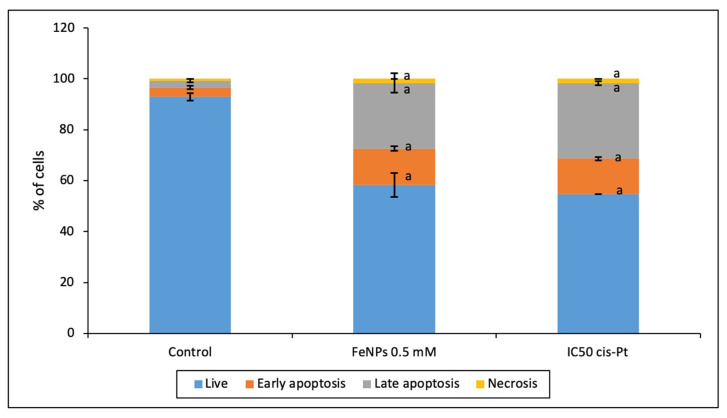
Evaluation of apoptosis/necrosis by flow cytometry. Results expressed as mean ± SEM. (a) vs. control (*p* < 0.05).

Figure 10 shows that the exposition to FeNPs reduced significantly the % of live cells respect untreated cells. Moreover, these nanoparticles increased the rate of apoptotic and necrotic cells at the present conditions with respect to control cells. There were not differences between ultra-small iron nanoparticles and IC_50_ of cis-platinum (cis-Pt).

## 4. Discussion

The application of nanoparticles in biomedical therapies will make possible the development of new diagnosis and treatment tools. One of the most promising aspect related to the use of nanoparticles in biomedicine therapy is its employ as carriers in gene therapy or drug delivery, due to its facility of synthesis, good biocompatibility and low toxicity [18]. In the present study, the uptake and distribution of the iron nanoparticles in the different compartments of colorectal cells were qualitatively visualized by TEM and quantified by ICP-MS. Although there is an increasing number of studies focused on understanding the interaction between the particles and the biological systems, publications about the mechanism of uptake and biodistribution are not yet clear [19]. The results of this study show that exposure of cells to ultra-small iron nanoparticles produced an increase in the levels of all the elements studied in the subcellular fraction corresponding to the mitochondria except copper (which seems to be directed towards the nucleus where an increase in its levels was detected). This traffic of elements towards this fraction could indicate their participation in the cellular processes involving mitochondria and lysosomes induced by iron nanoparticles that are detailed below.

The results revealed that the greatest abundance of iron at baseline levels resides in the subcellular fraction corresponding to the mitochondria. It is possible that FeNP went to the mitochondria fraction through the endosome-lysosome-autolysosome pathway, instead of going to mitochondria directly. After the exposure of the cells to the different treatments with NPs, the results showed that iron levels increased in all subcellular fractions, but especially in the fraction corresponding to the mitochondria, where levels increased 62 fold when the cells were exposed to FeNP, and 94 fold when they were exposed to Venofer^®^. These results are consistent with those obtained by TEM and with previous experiments that revealed a faster solubilization of Venofer^®^ than the synthetic FeNPs. Such solubilization might increase the bioavailability of ionic Fe to get into the mitochondria once the NPs have been internalized as such, as can be observed in Figure 1. It is known that iron is transported to the mitochondria, where it is utilized for synthesis of cofactors essentials for the function of enzymes involved in oxidation-reduction reactions, DNA synthesis and repair, and a variety of other cellular processes. Nowadays, the trafficking of iron to the mitochondria and normal mitochondrial iron metabolism, including heme synthesis and iron-sulfur cluster biogenesis, are being investigated [20].

There are different factors that could modify the clearance of this type of particles into the cells, like size, shape, or surface properties [21]. Recently, Feng and co-workers [7] studied the intracellular traffic of iron nanoparticles and the uptake in in vitro models. In their study, the iron nanoparticles were deposited in the cell structure within membrane-bound structures like lysosomes or endosome after the exposition, but not in mitochondria. This fact could be due to the exposition time of these experiments (2 h). In this way, the exposition time used in the present study (48 h) may explain a more compartmentalized distribution of this type of particles. On the other hand, the reduced size of the particles employed in the present study could promote the particles to cross the membranes and their transport to the mitochondria. Nevertheless, other authors that studied the intracellular traffic of other types of metallic nanoparticles, in particular, silver nanoparticles, suggested that they could be located close to the mitochondria [22,23].

At the present time, iron nanoparticles are being developed as an effective carrier in gene therapy [18]. In that way, some approaches based on metallic nanoparticles, like iron oxide nanoparticles [24] or gold silver alloy nanoparticles [25] have been employed to deliver genetic material to mitochondrial tissue. Therefore, ultra-small iron nanoparticles could be employed as a new tool due to their small size to be directed to the mitochondria.

Therefore, after confirming the nanoparticles localization, we evaluated the integrity of mitochondrial DNA and the respiratory activity of mitochondria. The effects of the FeNPs on mitochondria was elucidated measuring mtDNA copy number and deletions, and the respiratory activity. The ratio between the mtDNA genes ND4 and ND1 is considered as a marker of the so-called common deletion 20. This is because ND1 is in a very stable region of the mtDNA. Meanwhile, ND4 is in the middle of the region that suffer the common deletion [26]. mtDNA deletions may be considered as markers of mtDNA damage of oxidative origin or other [27]. In the present study, cells exposed to FeNPs and Venofer^®^ did not cause changes in this ratio, which can be interpreted as the absence of damage at the mtDNA. However, results showed a decrease in copy number of mtDNA. mtDNA copy number is considered as an indirect marker of mitochondrial function [28]. Also, some authors related the low copy number with the decrease of mitochondrial replicative activity [29] or with an increase in mitochondrial destruction for example through autophagy 11.

Results found in an mtDNA copy study were confirmed after analyzing mitochondrial respiration by Seahorse technology. In fact, Figure 5 shows how the ultra-small FeNPs affect maximal respiration and mitochondrial spare respiratory capacity, which is regarded as an important aspect of mitochondrial function and is defined as the difference between basal ATP production and its maximal activity. When cells are subjected to stress, energy demand increases, requiring more ATP to maintain cellular functions. A cell with a larger spare respiratory capacity can produce more ATP and overcome more stress, including oxidative stress [30]. Overall, changes found in mitochondrial function after nanoparticles administration present a very interesting opportunity for ultra-small iron nanoparticles as antiproliferative agents in cancer, since they would allow the reduction of metabolic activity in cancer cells when administered locally in the tumor, without causing alterations in mitochondrial DNA from adjacent healthy cells.

Then, from the study, it can be demonstrated that iron particles decreased mitochondria functionality without increasing mtDNA damage. This could be used to promote cell death using the mitochondria as a target, when necessary, without affecting healthy neighbor cells. Preserving the integrity of the mtDNA of adjacent cells is a very important advantage of this possible therapeutic tool when it is administered locally, since the strategy of producing cell death through oxidative damage is not very selective and could compromise neighboring cells. Some of these effects of nanomaterials have been proposed previously by other authors [9], but the effect on the respiratory function have not been elucidated yet. Consequently, the present work could contribute to understanding the relation between iron nanoparticles and mitochondrial function.

Autophagy is an intracellular degradation process associated with an appropriate replacement of certain types of cellular organelles, and it is important for the cell [12]. Some authors have proposed autophagy as one of the possible mechanisms of the nanoparticle mechanism of action in cells [31]. In that way, the exposition of different cell lines to metallic nanoparticles has been related to the activation of the autophagic process [4]. Certain authors [32] showed that rare-earth elements nanoparticles, samarium (Sm)/europium (Eu) and gadolinium (Gd)/terbium(Tb), induced severe autophagy in cervical cancer cells (HeLa). Sun and co-workers [33] demonstrated the autophagic process and the mitochondrial damage of silica particles in hepatocytes. The relation of autophagy in mitochondria has not been deeply studied. Yoo and co-workers [34] suggested that one link between autophagy and mitochondria is the process called mitophagy, the selective degradation of mitochondria by autophagy. By means of this process, the excess or defective mitochondria following damage or stress (i.e., oxidative stress) is removed. Damaged mitochondria cause a depletion in ATP and a release of cytochrome c, which leads to activation of caspases and onset of apoptosis. This process courses with the sequestration and hydrolytic degradation by lysosomes [35,36]. In our study, confocal microscopy results suggested a greater autophagic activity in FeNP-treated cells. This result agrees with those obtained from the TEM images.

Moreover, to determine if the autophagic flux was initiated by the FeNPs, p62 was determined. In that way, p62 decreased when cells were treated with nanoparticles producing a similar effect of rapamycin. In that way, p62 low levels and high levels of autophagy induction could suggest that the beginning of the autophagic process was mediated by the nanomaterials [37]. Consequently, these nanoparticles induced the autophagy in cells.

Furthermore, the ability of ultra-small iron nanoparticles to induce apoptosis/necrosis in colorectal tumoral cells was evaluated. Figure 10 shows that at the present conditions FeNPs induced mainly apoptosis and low necrosis levels at similar conditions that IC_50_ cis-platinum promoted. The role of iron nanoparticles in the cell death mechanism has been previously investigated by other authors that described that the rate of apoptosis induction depends on the size, form, and shape of nanomaterials [7]. Indeed, this effect could be mediated by the capacity of Fe to conjugate with ROS and affect cell membranes, proteins, or mitochondria [38].

Indeed, the results obtained showed that ultra-small nanoparticles could be used to promote cell death using the mitochondria as a target, when necessary, although the impact on healthy neighbor cells remains to be determined.

In a previous investigation from our research group [15], it was observed that the same FeNPs tested in this study increased ROS generation, which could lead to an increased mitochondrial autophagy as consequence of the increased oxidative damage at this organelle. This situation would cause the reported fall in mitochondrial respiration, as well as in glycolytic capacity. This sequence of events would be responsible for the entry of the cells into the quiescence state.

## 5. Conclusions

In conclusion, under the experimental conditions of this study, when the colorectal cells were exposed to the ultra-small iron nanoparticles tested, they reached the subcellular compartment corresponding to the mitochondria, quantitatively finding the highest levels of iron in the aforementioned compartment. Electron microscopy studies showed the presence of autolysosomes after 48 h of exposure, compatible with the mitophagy process. Exposure to ultra-small iron nanoparticles did not cause deletions in mtDNA, but a reduction in the number of mtDNA copies, indicative of a reduction in the number of mitochondria in these tumor cells. Mitochondrial functionality studies demonstrated a reduction in respiration parameters and a decrease in cellular glycolytic metabolic activity, which are the logical consequence of the reduction in the number of mitochondrial copies. Treatment with synthesized ultra-small iron nanoparticles resulted in the migration of the cellular state towards quiescence. Finally, autophagy studies using confocal fluorescent microscopy corroborated the images obtained by electron microscopy, reinforcing the presence of the mitophagy process. All these results make ultra-small iron nanoparticles a relevant tool for use as a therapeutic agent in mitochondria-dependent diseases, such as cancer.

## Figures and Tables

**Table 1:** Metal concentrations in HT-29 subcellular fractions.

Subcellular Fraction	Group	Na ng/10^6^ Cells	Mg ng/10^6^ Cells	P ng/10^6^ Cells	K ng/10^6^ Cells	Ca ng/10^6^ Cells	Fe ng/10^6^ Cells	Cu ng/10^6^ Cells	Mn ng/10^6^ Cells	Zn ng/10^6^ Cells	Se ng/10^6^ Cells
Mitochondria + Peroxisome	Control	1353.3 ± 60.1	115.8 ± 2.8	7821.7 ± 1188.7	942.5 ± 125.6	500.1 ± 24.2	28.2 ± 5.1	4.1 ± 2.3	1.5 ± 0.01	49.9 ± 7.2	0.084 ± 0.01
	Fe NPs	1690.0 ± 471.5 ^a^	423.8 ± 9.5 ^a^	18058.6 ± 525.2 ^a^	1364.8 ± 305.3 ^a^	985.7 ± 119.0 ^a^	1345.2 ± 370.1 ^a^	2.8 ± 0.3	1.2 ± 0.01 ^a^	72.0 ± 4.3 ^a^	0.227 ± 0.03 ^a^
	Venofer^®^	1915.6 ± 6.6 ^a,b^	432.2 ± 37.3 ^a^	18204.3 ± 463.9 ^a^	1635.4 ± 584.6	1043.1 ± 12.5	2636.6 ± 27.8 ^a,b^	3.7 ± 0.4 ^b^	1.8 ± 0.02 ^b^	71.0 ± 2.1	0.308 ± 0.02 ^a^
Cytosol + Mb + Golgi + REP	Control	3561.4 ± 687.1 ^c^	352.1 ± 43.9 ^c^	4531.3 ± 445.2	4307.3 ± 805.6 ^c^	386.8 ± 15.8 ^c^	8.6 ± 0.04 ^c^	0.4 ± 0.2	1.3 ± 0.2	43.3 ± 0.69	0.043 ± 0.01 ^c^
	Fe NPs	4193.0 ± 471.5 ^e^	391.4 ± 9.5	5885.9 ± 525.2 ^e^	4918.1 ± 305.3 ^e^	496.7 ± 119.0 ^e^	300.9 ± 37.8 ^a,e^	0.3 ± 0.2 ^e^	1.1 ± 0.2	45.4 ± 4.3 ^e^	0.060 ± 0.03 ^e^
	Venofer^®^	4064.2 ± 193.1 ^g^	451.8 ± 37.3	6579.2 ± 463.9 ^a,g^	5359.3 ± 584.6 ^g^	435.4 ± 12.5 ^a,g^	388.2 ± 27.8 ^a,b,g^	0.6 ± 0.1 ^g^	1.6 ± 0.2	49.9 ± 2.1 ^a,g^	0.104 ± 0.02 ^a,g^
Nucleus	Control	898.6 ± 128.9 ^c,d^	81.2 ± 32.7 ^d^	5112.6 ± 3982.1	299.2 ± 137.7 ^c,d^	717.1 ± 366.9	18.9 ± 7.3	2.0 ± 0.6	2.5 ± 0.1 ^d^	77.6 ± 18.4	0.213 ± 0.01 ^c,d^
	Fe NPs	942.7 ± 11.7 ^e,f^	123.5 ± 9.9 ^e,f^	8480.4 ± 873.7 ^e,f^	433.0 ± 16.5 ^e,f^	825.0 ± 93.0 ^f^	454.41 ± 98.3 ^a,e^	4.3 ± 0.6 ^a,e,f^	4.0 ± 0.9 ^e,f^	44.2 ± 5.1 ^e^	0.102 ± 0.01 ^a,e^
	Venofer^®^	1046.2 ± 64.8 ^g,h^	180.8 ± 26.4 ^a,b,g,h^	11586.2 ± 600.0 ^b,g,h^	657.4 ± 126.9 ^b,g,h^	978.6 ± 133.2 ^h^	1271.1 ± 156.8 ^a,b,g,h^	5.6 ± 0.7 ^a,g,h^	4.4 ± 0.9 ^g,h^	72.0 ± 25.3	0.175 ± 0.05

Statistics between groups within each subcellular fraction: ^a^ vs. control; ^b^ vs. Fe NPs; Statistics in each group among the different subcellular fractions; ^c^ vs. control group of the subcellular fraction corresponding to Mitochondria + Peroxisome; ^d^ vs. control group of the subcellular fraction corresponding to Cytosol + Mb + Golgi + REP; ^e^ vs. Fe NPs group of the subcellular fraction corresponding to Mitochondria +Peroxisome; ^f^ vs. Fe NPs group of the subcellular fraction corresponding to Cytosol + Mb + Golgi + REP; ^g^ vs. Venofer^®^ group of the subcellular fraction corresponding to Mitochondria +Peroxisome; ^h^ vs. Venofer^®^ group of the subcellular fraction corresponding to Cytosol + Mb + Golgi + REP.

## Data Availability

The data presented in this study are available on request from the corresponding author. The data are not publicly available due to privacy.
